# Integrated Analysis of lncRNAs and mRNAs Reveals Complex Gene Network Mediated by lncRNAs and Regulatory Function of *MuLRR-RLK-AS* in Response to Phytoplasma Infection in Mulberry

**DOI:** 10.3390/biom14030308

**Published:** 2024-03-05

**Authors:** Zixuan Liu, Chaorui Liu, Teng Zhao, Lulu Yang, Qiqi Shang, Gefan Wang, Zhaoyang Liu, Yingping Gai, Xianling Ji

**Affiliations:** 1 College of Forestry, Shandong Agricultural University, Taian 271018, China; 2022120331@sdau.edu.cn (Z.L.); 2021010051@sdau.edu.cn (C.L.); 2022110256@sdau.edu.cn (L.Y.); 2022110255@sdau.edu.cn (Q.S.); 2022120341@sdau.edu.cn (G.W.); lzysdau@163.com (Z.L.); 2 College of Life Sciences, Shandong Agricultural University, Taian 271018, China; 2021110673@sdau.edu.cn

**Keywords:** mulberry, phytoplasma, lncRNA, LRR receptor-like serine/threonine-protein kinase

## Abstract

Phytoplasma disease is one of the most serious infectious diseases that affects the growth and development of mulberry. Long non-coding RNAs (lncRNAs) play an important role in plants’ defense systems; however, the contribution of lncRNAs in the response to phytoplasma infection in mulberry is still largely unknown. Herein, strand-specific RNA sequencing was performed to profile the mRNAs and lncRNAs involved in the response to phytoplasma infection in mulberry, and a total of 4169 genes were found to be differentially expressed (DE) between healthy and phytoplasma-infected leaves. Moreover, 1794 lncRNAs were identified, of which 742 lncRNAs were DE between healthy and infected leaves. Target prediction showed that there were 68 and 44 DE lncRNAs which may function as cis and trans-regulators, targeting 54 and 44 DE genes, respectively. These DE target genes are associated with biological processes such as metabolism, signaling, development, transcriptional regulation, etc. In addition, it was found that the expression of the antisense lncRNA (*MuLRR-RLK-AS*) of the leucine-rich repeat receptor-like protein kinase gene (*MuLRR-RLK*) was decreased in the phytoplasma-infected leaves. Interestingly, it was found that overexpression of *MuLRR-RLK-AS* can inhibit the expression of *MuLRR-RLK*. Moreover, it was found that the expression levels of PTI-related and MAPK genes in the transgenic *MuLRR-RLK* Arabidopsis plants were significantly higher than those in the wild-type plants when inoculated with pathogens, and the transgenic plants were conferred with strong disease resistance. Our results demonstrate that *MuLRR-RLK-AS*, as a trans-regulatory factor, can inhibit the expression of the *MuLRR-RLK* gene and is a negative regulatory factor for mulberry resistance. The information provided is particularly useful for understanding the functions and mechanisms of lncRNAs in the response to phytoplasma infection in mulberry.

## 1. Introduction

As the only feed tree species of silkworm and an important material basis of sericulture, mulberry has been cultivated for thousands of years in China [1]. Moreover, the leaves, fruits, branches, bark, and roots of mulberry are also of high edible, medicinal, and feeding value [2]. In addition, as an ecological tree species, mulberry also plays an important role in sand prevention and control, rocky desertification control, saline alkali land control, soil and water conservation, and returning farmland to forest, and its economic and ecological value has been paid more and more attention [3]. However, this important economic and ecological tree is susceptible to many pathogens and pests, and more than 100 mulberry diseases have been found, among which is mulberry yellowing dwarf disease, caused by phytoplasma; this disease is not only rapidly infectious, but also explosive and destructive, and often leads to the destruction of large mulberry gardens, which seriously limits the realization of the feeding, medicinal, and ecological value of mulberry trees [4,5,6]. In recent years, with the rapid development of molecular plant pathology and molecular biology, the research on the mechanism of phytoplasma disease has gradually deepened, and a large number of studies have been carried out to analyze mulberry defense responses at transcript, protein, metabolite, and epigenetic levels [6,7,8,9,10]. However, the information obtained is still insufficient to reveal the molecular mechanism underlying mulberry phytoplasma disease.

Plants have evolved a set of elaborate strategies for coping with pathogen infection. In addition to protein-coding genes, which have important regulatory role as key regulators in plant defense responses, increasing evidence shows that some non-coding RNAs (ncRNAs) also play an important role in plant immune response and are important elements of gene regulation and plant resistance mechanisms [11]. lncRNAs are a diverse class of RNA molecules more than 200 nucleotides in length and have no protein-coding capacity [12]. In recent years, with the rapid development of high-throughput sequencing, a large number of lncRNAs have been identified from various plants, and many lncRNAs have been functionally characterized in some plants [13,14,15,16,17,18,19,20,21]. It has been shown that lncRNAs could act in a cis or trans fashion to control gene expression at transcriptional and post-transcriptional levels, executing as signals, decoys, guides, or scaffolds [22,23], and many studies have shown that lncRNAs can act as “competing endogenous RNA” (ceRNA) by competing with mRNAs to bind to miRNAs and changing the expression of the miRNA target genes [24]. Some lncRNAs have been suggested to play important regulatory roles in plant defense systems and to be involved in responses to viral, fungal, and bacterial infections in plants [14,25,26,27,28,29,30,31,32,33,34,35,36,37,38,39,40]. Phytoplasma is an unculturable obligate parasitic pathogen, and despite the fact that several potential virulence factors have been discovered recently, its pathogenic mechanism is still unknown [41,42]. Studies examining plant–phytoplasma interactions have reported that a large number of non-coding RNAs were regulated following infection with phytoplasma. For example, many miRNAs associated with phytoplasma infection have been identified in mulberry [8,10], and some lncRNAs involved in phytoplasma infection have been recognized in *Paulownia tomentosa* [43,44]. It was suggested that lncRNAs may play an important role in plant–phytoplasma interactions. However, few lncRNAs have been experimentally tested for their biological functions in plant–phytoplasma interactions. Furthermore, it is evident that lncRNAs have less evolutionary conservation across species and lower expression levels than protein-coding genes but have a higher degree of time- or tissue-specific expression patterns [21,45]. Therefore, the existing information is still insufficient to understand the molecular mechanisms of lncRNAs involved in plant–phytoplasma interactions. The identification and characterization of species-specific phytoplasma-responsive lncRNAs is of great significance.

In the present study, strand-specific RNA transcriptome sequencing was performed to profile the lncRNAs involved in the response of mulberry to phytoplasma infection, and the differentially expressed lncRNAs were identified and their functions were discussed. Moreover, the functions of the antisense lncRNA (*MuLRR-RLK-AS*) of the leucine-rich repeat receptor-like protein kinase gene (*MuLRR-RLK*) were analyzed. The information provided will provide important clues for revealing insights into the roles of lncRNAs in mulberry–phytoplasma interactions and better understanding of the molecular mechanisms of phytoplasma pathogenicity.

## 2. Materials and Methods 

### 2.1. Plant Materials and Growth Conditions

Mulberry seedlings (*Morus multicaulis* Perr.) were grafted with phytoplasma-infected scions to inoculate them with phytoplasma, and the grafted seedlings showing phytoplasma disease symptoms, such as stunting, witches’-broom, and yellowing of the leaves, were confirmed by PCR amplification of the 16S rRNA gene of phytoplasma with the primers TAAAAGACCTAGCAATAGG and CAATCCGAACTGAGACTGT to confirm successful inoculation with phytoplasma as described previously [46]. All the healthy and infected mulberry seedlings were cultivated in a greenhouse under a light/dark regime (16 h light, 100 μmol·m^−2^·s^−1^ photon flux density/8 h dark) at 25 °C ± 1 °C with a humidity of 50–60%. *Arabidopsis thaliana* (Col-0) and *Nicotiana benthamiana* seedlings were cultured in incubators at 22 °C and 24 °C, respectively, under a light/dark regime (16 h light, 100 μmol·m^−2^·s^−1^ photon flux density/8 h dark) and humidity of 50–60%. 

### 2.2. Construction of cDNA Libraries and High-Throughput Sequencing 

Total RNA was extracted from the leaves of healthy and phytoplasma infected mulberry seedlings using the RNA extraction kit (Invitrogen Corporation, Carlsbad, CA, USA) and then treated with Epicentre’s Ribo-Zero™ kit (Epicentre, Madison, Wisconsin, USA) to deplete the ribosome RNA. The quality of the RNA was confirmed using the Agilent 2100 Bioanalyzer (Agilent Technologies, Santa Clara, CA, USA) (2100 RIN C7.0 and 28S/18S C0.7). Thereafter the strand-specific libraries were constructed using the Illumina TruSeq Stranded RNA Kit (Illumina, San Diego, CA, USA) and sequenced on an Illumina HiSeq 2000 sequencer at a depth of 10 million reads per library. Raw sequencing data were filtered to discard the adapter contaminations, shorter fragments < 200 bp, and low-quality tags (Q20 < 20). Then, the clean reads obtained were merged and aligned to the *M. notabilis* reference genome (https://www.ncbi.nlm.nih.gov/genome/?term=Morus; accessed on 24 June 2013) sequence using the spliced read aligner TopHat, and the mapped reads were assembled de novo using Cufflinks (2.0.2) to construct transcript sequences.

### 2.3. Identification of lncRNAs and Target Prediction

Assembled transcripts were then compared with the reference genome using Cuffcompare program from the Cufflinks package (2.0.2). According to the annotation of the *M. notabilis* genome sequence, unknown transcripts other than those identified as known protein coding transcripts were used to screen for putative lncRNAs. First, the assembly transcripts were compared with the reference transcripts, and only the five kinds of class transcripts, including a transfrag, that completely fall within a reference intron, unknown intergenic transcripts, exonic overlapping on the antisense chain of a reference transcript, and generic exonic overlapping with a reference transcript, were retained and defined as “i”, “u”, “x”, and “o”, respectively. Second, the transcripts with a length > 200 bp and an exon count exceeding one were saved for the next step. Finally, lncRNAs were screened using four analytical tools, namely, the Coding Potential Calculator (CPC) (https://cpc.cbi.pku.edu.cn/) (with e-value “1 × 10^−10^”), the Coding-Non-Coding Index (CNCI) (https://github.com/www-bioinfo-org/CNCI), PLEK v1.2 (https://sourceforge.net/projects/plek/), and Pfam (https://pfam.xfam.org) (E 0.001–domE 0.001), to screen these ncRNAs for RNA length > 200 nt and ORF < 50aa.

To understand biological function of the DE lncRNAs, their potential target genes were predicted based on cis-acting and trans-acting modes. Based on the genomic location of the lncRNAs and protein coding genes, the protein coding genes within 5 kb upstream or downstream of the lncRNA were selected, and if their Pearson correlation coefficient with lncRNA was ≥0.8, they were selected as potential target genes of the lncRNAs. If a protein coding gene was on the antisense strand of the lncRNA and had a complementary sequence > 50 nt with the lncRNA and the correlation between the protein coding gene and lncRNA was ≥0.8, the protein coding gene was predicted as the potential target gene for the lncRNAs that act in trans-acting mode.

### 2.4. Gene Expression Analysis and qRT-PCR Validation

The expressions values of genes were calculated using the RPKM (reads per kb per million reads) method, and statistical analysis was carried out according to Poisson distribution [44]. Only the genes with expression values which changed more than twice (*p* ≤ 0.05) between healthy and phytoplasma-infected leaves were designated as significantly differentially expressed genes. To verify the reliability of the high-throughput analysis results, some mRNAs and lncRNAs were randomly selected following the principle of random sampling for qRT-PCR analysis, and the qRT-PCR was carried out on the CFX96^TM^ real-time machine (Bio-Rad) using SYBR Green Premix Ex Taq TM (Takara Biotechnology, Tokyo, Japan). Then, the expression of the gene was normalized to *EF1-a* transcript abundance and calculated using the 2^−ΔΔ^CT method [47], and the samples were analyzed in triplicate.

### 2.5. Gene Ontology Analysis

Assembled transcripts were used as query sequences for BLASTN searches against the reference *M. notabilis* database with online tools (https://morus.swu.edu.cn/morusdb/blast) to obtain the gene IDs for the transcripts, and gene ontology (GO) analysis was performed based on the gene IDs obtained using the online tool (https://morus.swu.edu.cn/morusdb/searchgo) with the ANNEX annotation augmentation function (version 2.3.1) according to the method described before [48,49].

### 2.6. Gene Cloning and Phylogenetic Analysis

Reverse transcriptase M-MLV (Promega) was used to synthesize cDNA with the RNA isolated, and then the cDNA obtained was used for PCR amplifications with the specific primers (for *MuLRR-RLK*: ATGGCCAAACTAAGCCTCCTACT; CTAAGCTCTTCTCCTTCTCCGT; for *MuLRR-RLK-AS*: TGAGTTTTTGCATCGATGATGGGA; GTGCCTTCCCATCGCCATCGTT) designed based on the nucleotide sequence of the gene. Then, the target DNA fragment was recovered from the PCR products by electrophoresis and subcloned into the pMD18-T vector (Takara, Dalian, China) and sequenced. The multiple alignments between the amino acid sequences encoded by genes and the homologous proteins from other plants were performed using the DNAMAN program. The phylogenetic tree of the proteins from diverse species was generated with the neighbor-joining method in the MEGA program (version 6.0) with 1000 bootstraps. Modeling of the three-dimensional structure of the protein was performed using the SWISS-MODEL server.

### 2.7. Subcellular Localization

To observe the subcellular localization of the target protein, the gene coding the protein was fused with the green fluorescent protein gene (GFP) and was cloned into the binary vector pBI121 under the control of 35S. The Agrobacteria containing the vector constructed above infiltrated the *N. benthamiana* leaf epidermal cells. Then, the small sections of the infiltrated leaves were excised 48 to 72 h after infiltration using a Bio-Rad MRC1024 confocal laser scanning microscope (Bio-Rad, Hemel Hempstead, UK) with an excitation wavelength of 488 nm and an emission wavelength of 505–530 nm at 400× magnification.

### 2.8. Promoter Activity Analysis

The specific primers were designed based on the *M. notabilis* genome (https://www.ncbi.nlm.nih.gov/genome/?term=Morus; accessed on 24 June 2013) to amplify a 2000 bp upstream region of the *MuLRR-RLK* (named *pMuLRR-RLK*) or *MuLRR-RLK-AS* gene (named *pMuLRR-RLK-AS*). Then, the 35S promoter in the binary vector pBI121 was replaced by the promoters, respectively, to create the expression vector pMuLRR-RLK::GUS or pMuLRR-RLK-AS::GUS. The vector created was then transferred into Agrobacterium strain GV3101, which was used to infiltrate tobacco leaves, and the GUS expression in the infiltrated leaves was assessed by histochemical staining [50].

### 2.9. Production of Transgenic Arabidopsis

For production of transgenic Arabidopsis, MuLRR-RLK or MuLRR-RLK-AS was introduced into the expression vector pBI121 under the control of the 35S promoter, respectively, to construct their expression vectors. Meanwhile, both the MuLRR-RLK and MuLRR-RLK-AS genes were ligated into the same modified expression vector pLGNL under the control of the 35S promoter, respectively, to construct their co-expression vector. Then, the vectors constructed were introduced into A. tumefaciens strain GV3101, which was used to transform Arabidopsis with the floral dip method [51]. The seeds of transgenic plants were selected, and the T3 generation transgenic seeds were used for subsequent experiments.

### 2.10. Plant Treatment

In the case of exogenous SA treatments, 5 mmol·L^−1^ SA solution was evenly sprayed onto the adaxial surfaces of mulberry leaves. The CFU of *Pseudomonas syringae* pv. tomato DC3000 (*Pst*. DC3000) assay was performed with the serial dilution method, and the bacterial suspensions (10^5^ CFU·mL^−1^) of *Pst.* DC3000 were injected into the rosette leaves of Arabidopsis seedlings to perform inoculation. All of the inoculated seedlings above and the control mulberry seedlings sprayed or injected with sterilized water were incubated in a humidified 95% chamber for 48 h to ensure successful inoculation. The rosette leaves of Arabidopsis were detached, the adaxial surface of the leaves was inoculated with the mycelium plugs (Φ 2 mm) from the actively growing *Botrytis cinerea* colonies, and the leaves inoculated with PDA medium plugs (Φ 2 mm) were used as controls. All of the inoculated leaves and controls were placed in covered Petri dishes, and the disease severity was examined daily. All the treatments were conducted independently at least three times.

### 2.11. Detection of Colony-Forming Units

The leaves inoculated with *Pst.* DC3000 were ground in sterile water, and the suspension was serially diluted in a ten-fold series in sterile water. Then, 100 μL of each dilution was spread-plated onto King’s B medium and incubated at 28 °C. After 48 h of incubation, colony forming units were counted. All of the experiments were independently repeated at least three times.

### 2.12. Statistic Analysis

For the high-throughput sequencing analysis, three biological replicates with healthy and infected leaves were used for strand-specific transcriptome analysis. For gene function, expression, and other physiology and biochemistry assays, all the experiments were bioreplicated at least three times. The results were analyzed by analysis of variance, and when the *p*-values were less than 0.05, the difference was considered significant.

## 3. Results

### 3.1. High-Throughput Sequencing and DEG Analysis

**Figure 1:**
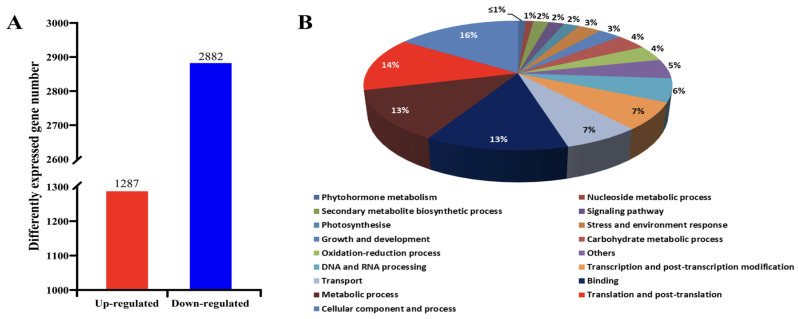
Statistics of the differentially expressed genes and their distribution percentage in different categories. (**A**) Statistics of the differentially expressed genes (DEGs); (**B**) gene ontology (GO) analysis of the DEGs.

Using the Illumina platform, the strand-specific transcriptome libraries of healthy (HL) and phytoplasma-infected mulberry leaves (IL) were sequenced, and a total of 36.0 and 37.5 million clean reads were obtained in the HL and IL libraries, respectively. After calculating the expression level (FPKM) of each gene, a total of 4169 genes were found to be differentially expressed between HL and IL libraries, among which 1287 genes were up-regulated and 2882 genes were down-regulated in the IL libraries (Figure 1A). All of the differentially expressed genes (DEGs) detected are shown in Supplementary Table S1.

Through gene ontology (GO) analysis of the DEGs, they were classified into 17 functional categories (Figure 1B). The first category of genes was associated with cellular components and processes, the genes involved in translation and post-translation modification belonged to the second category, and the third category included the genes related to metabolic processes. Interestingly, it was found that 3% and 2% of the DEGs detected were associated with stress and environment response and signaling pathways, respectively. The other categories of DEGs included transcription and post-transcription modifications, carbohydrate metabolic processes, growth and development, etc. This indicates that the regulatory network of the genes in response to phytoplasma infection is complex in mulberry. The detailed functional GO terms of the DEGs are provided in Supplementary Table S1.

**Figure 2:**
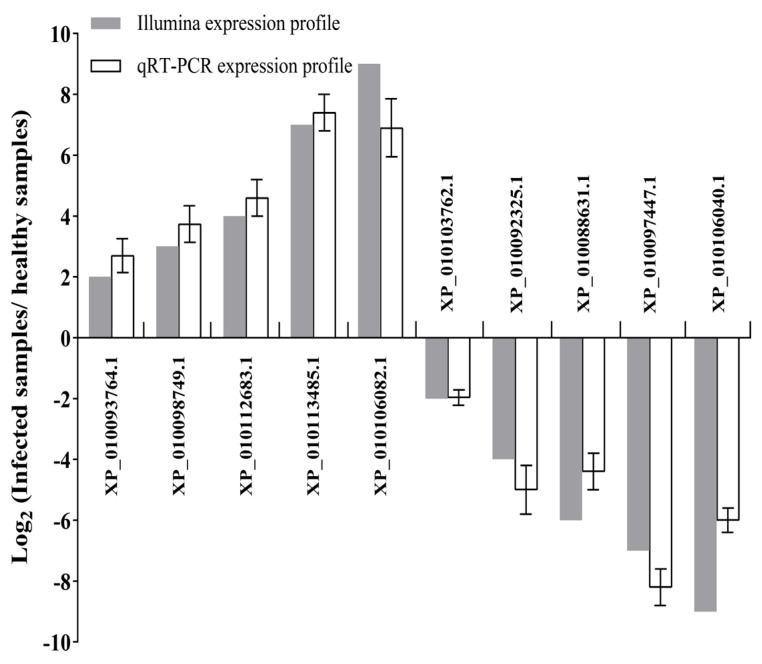
Verification of the DEG expression patterns by qRT−PCR. The *EF1-α* was used as a reference gene to evaluate the relative expression levels of the selected genes with the 2^−ΔΔCt^ method. The efficiency of all the qRT−PCR reactions was 95%~105%. The column indicates the log_2_ ratio of the relative expression levels of the genes of phytoplasma−infected leaves versus healthy leaves, and values are reported as means ± SD.

To verify the reliability of the gene expression profiles obtained by RNA-seq, 10 DEGs including the up-regulated and down-regulated genes responsive to phytoplasma infection were randomly selected for qRT-PCR validation (Figure 2). The qRT-PCR results showed that the expression levels of all the selected genes show similar change profiles between qRT-PCR and RNA-seq results, indicating that the results obtained by RNA-seq are reliable.

### 3.2. Identification and Characterization of lncRNAs

**Figure 3:**
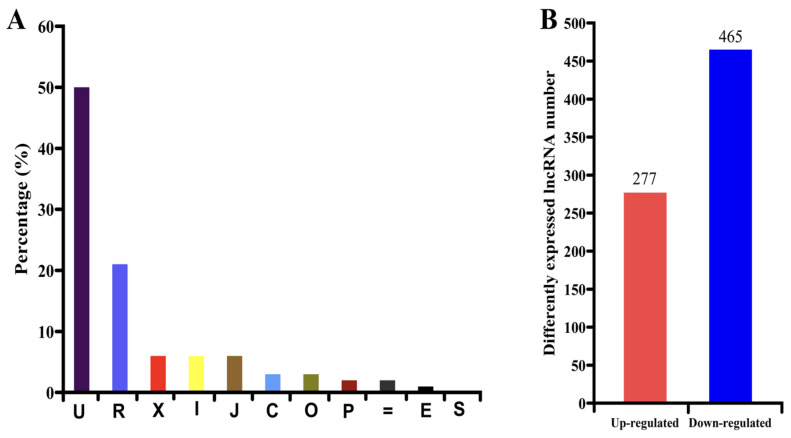
Categories of lncRNAs obtained (**A**) and statistics of differentially expressed lncRNAs (DELs) (**B**). ‘U’ represents an unknown intergenic transcript; ‘R’ represents a repeat sequence which was determined to refer to the soft-masked reference sequence and applied to transcripts with at least 50% lower case; ‘X’ represents an exonic overlap with a known transcript on the opposite strand; ‘I’ indicates a transfrag that entirely falls within an intron of a reference transcript; ‘J’ represents a potential novel isoform (fragment) which has at least one splice junction shared with a reference transcript; ‘C’ represents contained; ‘O’ represents an lncRNA that has generic exonic overlap with a known transcript; ‘P’ represents a possible polymerase run-on fragment that is within 2K bases of a reference transcript; ‘=’ represents complete match with the intron chain; ‘E’ indicates single-exon transfrag overlapping of an exon and at least 10 bp of an intron of a reference gene, indicating that it may be a pre-mRNA fragment; ‘S’ represents an intron of the transfrag overlapping with an intron of a known transcript on the opposite strand.

In addition to the above mRNAs, a large number of lncRNAs were also identified. In total, 1794 unique lncRNAs were obtained from the HL and IL libraries. Based on the locations of the lncRNAs obtained in the genome, 50.5% of the lncRNAs were located in the intergenic regions, 7.0% of the lncRNAs were exonic overlaps with known transcripts on the opposite strand, 7.0% of them were transfrags falling entirely within intron of known transcripts, and 6.9% of them were potentially novel isoforms (Figure 3A). To identify phytoplasma-responsive lncRNAs, the expression levels of the lncRNAs in HL samples and IL samples were evaluated. Of these 1794 lncRNAs, 742 lncRNAs were differentially expressed between HL samples and IL samples, including 277 up-regulated lncRNAs and 465 down-regulated lncRNAs (Figure 3B and Supplementary Table S2).

**Figure 4:**
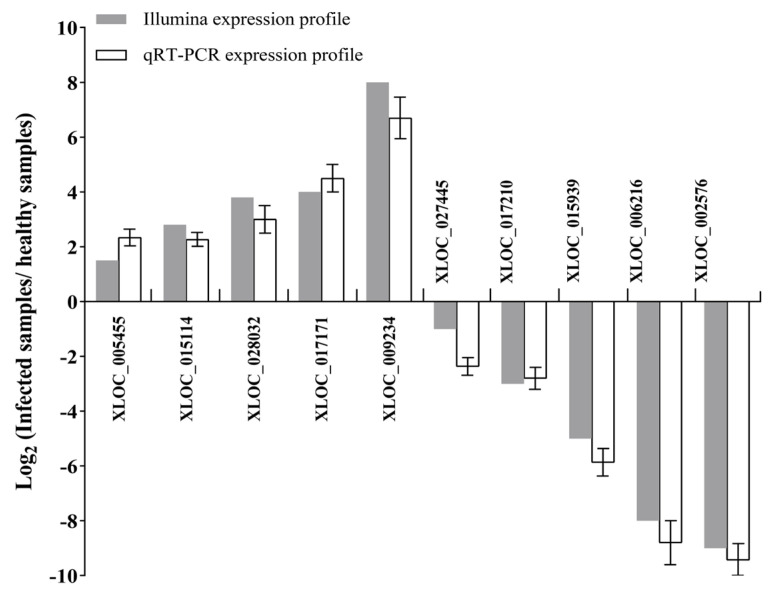
Verification of the expression patterns of the differentially expressed lncRNAs by qRT−PCR. Using EF1-α as a reference gene, the expression levels of the selected lncRNAs were analyzed with the 2^−ΔΔCt^ method. The efficiency of all the qRT−PCR reactions was 95%~105%. The column indicates the log_2_ ratio of the relative expression levels of the lncRNAs of phytoplasma−infected leaves versus healthy leaves, and values are reported as means ± SD, *n* = 3 in each group.

In order to confirm the reliability of the expression patterns of the lncRNAs obtained, the expression levels of 10 randomly selected lncRNAs were analyzed by qRT−PCR. The results showed consistent results between RNA-seq and qRT−PCR data (Figure 4), suggesting that the expression patterns of lncRNAs obtained are highly reliable, and the DE lncRNAs selected may play major roles in response to phytoplasma infection in mulberry.

### 3.3. Target Prediction of the Differentially Expressed lncRNAs (DELs)

Previous studies have shown that lncRNAs are preferentially located in the genomic regions adjacent to the genes they regulate. In order to reveal the potential functions and regulatory mechanism of lncRNAs in response to phytoplasma infection, firstly, the protein-coding genes located within <2 kb from the lncRNAs were predicted. A total of 621 genes were predicted as target genes for the 1122 lncRNAs, of which 465 were differentially expressed lncRNAs (DELs). Further analysis showed that 54 DEGs were potential target genes of 68 DELs, of which 13 were up-regulated and 41 were down-regulated in the infected leaves. Interestingly, it was found that the lncRNAs and their target genes not only exhibit the same expression trend, but also exhibit opposite expression trends. In addition, it was found that some target genes could be targeted by multiple lncRNAs, indicating that the regulatory mechanism of lncRNAs is complex (Table 1 and Supplementary Table S3). GO term analyses showed that these differentially expressed targeted genes were enriched in diverse biological processes, such as the metabolic process, development, defense responses, etc. (Table 1 and Supplementary Table S3). Therefore, these DELs may participate in the response to phytoplasma infection through various pathways by regulating the expression of their target genes.

Among the lncRNAs obtained, some are nature antisense transcripts (lncNATs) that were transcribed from the opposite strand of protein-coding regions and may regulate the expression of their sense transcripts. From our data, 378 lncNATs were identified and found to overlap with 365 protein-coding genes at their opposite strands. Among these lncNATs, there were only 44 differentially expressed lncNATs whose target genes were also differentially expressed. Furthermore, it was found that there were only nine lncNATs and their target genes showing the opposite expression tendency during phytoplasma infection (Table 2 and Supplementary Table S4). These lncNATs may also participate in the response to phytoplasma infection in mulberry by regulating the expression of corresponding genes.

### 3.4. Characterization of MuLRR-RLK-AS Trans-Target Gene MuLRR-RLK

**Figure 5:**
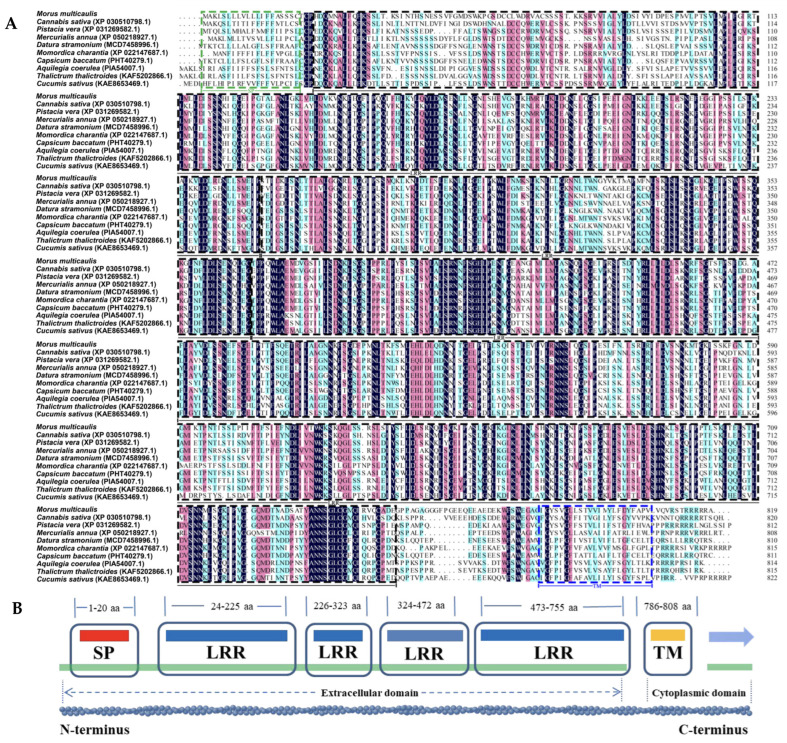
Alignment of the deduced amino acid sequences of MuLRR-RLK protein with other plant LRR-RLKs (**A**) and the domain organization of MuLRR-RLK (**B**). The identical amino acid residues are displayed in black, while the amino acids with >75% similarity are shaded in red and the amino acids with >50% similarity are shaded in blue. The heavy point indicates the conserved domain. SP, signal peptide; LRR, leucine-rich repeats; TM, transmembrane region; KD, Ser/Thr kinase domain.

Our data showed that the expression level of lncNAT XLOC_027445 (*LRR-RLK-AS*) was decreased, while the expression level of its trans-target gene, the putative LRR receptor-like serine/threonine-protein kinase gene (*LRR-RLK*), was increased in the phytoplasma-infected leaves, and these results were confirmed by qRT-PCR (Figure 2 and Figure 4). As we have seen, the *LRR-RLK* gene has not been reported to be associated with phytoplasma infection, and the gene in mulberry has not been characterized. To explore the roles of the *LRR-RLK* and *LRR-RLK-AS* in the response to phytoplasma infection in mulberry, the *LRR-RLK* gene was cloned from mulberry (*M*. *multicaulis* Perr.) and designated as *MuLRR-RLK*. The *MuLRR-RLK* gene encodes a protein of 819 amino acid residues, with a predicted molecular weight of 89.923 kDa and a *p*I of 5.98. Multiple sequence alignment analysis showed that the amino acids of MuLRR-RLK protein have high sequence identity with other plant LRR-RLK proteins, indicating that MuLRR-RLK is an evolutionarily conserved protein (Figure 5A). Protein structure analysis showed that MuLRR-RLK has structural characteristics of the LRR-RLK protein family, including a signaling peptide (SP) (1–20aa), four leucine-rich repeats (LRR) (112–173aa; 210–269aa; 520–577aa; 658–716aa) at the N-terminus, a transmembrane (TM) domain (784–809aa), and an intracellular Ser/Thr kinase domain (KD) at the C-terminus (Figure 5B).

**Figure 6:**
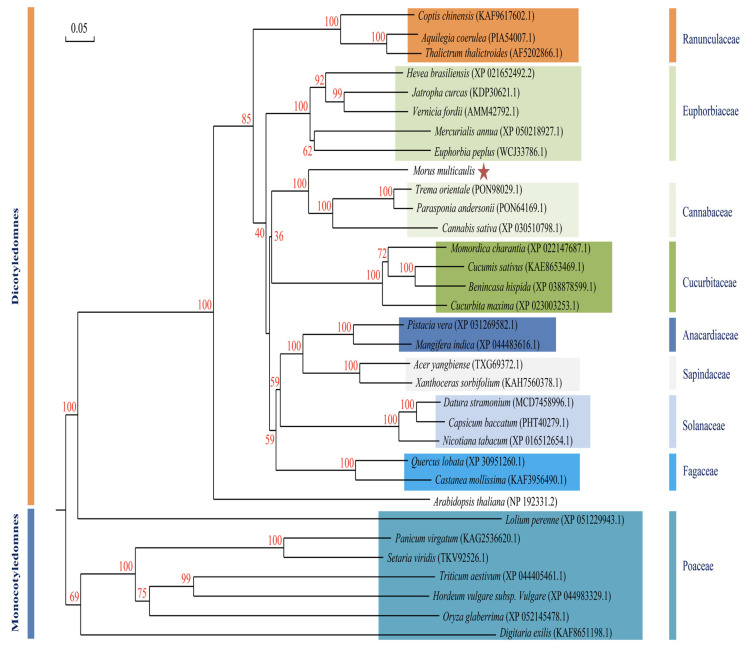
Phylogenetic analyses of MuLRR-RLK proteins from different plants. The phylogenetic tree was generalized using the neighbor-joining method. The numbers on the nodes are bootstrap values, and the scale indicates genetic distance. GenBank accession numbers of the proteins are shown in brackets. Red star indicates MuLRR-RLK protein.

**Figure 7:**
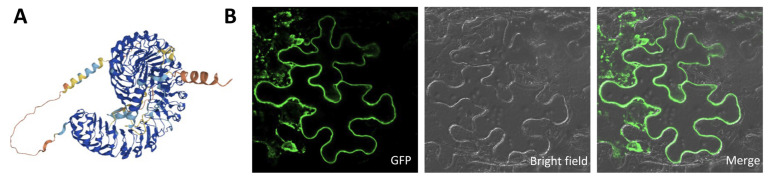
Predicted three-dimensional structure (**A**) and subcellular localization of MuLRR-RLK (**B**). MuLRR-RLK-GFP fusion protein was transiently expressed in *Nicotiana benthamiana* leaf epidermal cells and visualized with a confocal laser scanning microscope (Zeiss LSM880, Zeiss, Jena, Germany). The left image shows the cell with a GFP signal, and the bright-field view of the same cells is shown in the middle image. The right image indicates the overlays of the fluorescent and bright-field images.

Phylogenetic analysis of MuLRR-RLK and LRR-RLKs from other plants showed that the closest homology was between MuLRR-RLK and LRR-RLKs from *Trema orientale* and *Cannabis sativa* (Figure 6). Subcellular localization analysis showed that MuLRR-RLK localized to the plasma membrane as other LRR-RLK proteins (Figure 7), suggesting it may have similar roles as other LRR-RLK proteins which play important roles in perceiving and transmitting signals arising from different environmental conditions.

### 3.5. Expression Patterns of MuLRR-RLK and MuLRR-RLK-AS

**Figure 8:**
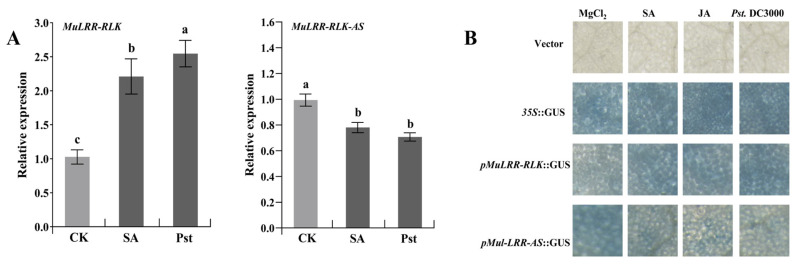
Induced expression profiles of *MuLRR-RLK* and *MuLRR-RLK-AS*. (**A**) Expression profiles of *MuLRR-RLK* and *MuLRR-RLK-AS* in mulberry leaves induced by *Pst.* DC3000 or SA. *EF1-α* was chosen as a reference gene for the qRT−PCR analysis, data represent the mean values of three biological replicates ± standard deviation, and different letters above the columns indicate significant differences at the 5% level according to the Duncan’s multiple range tests. Different letters indicate significant differences at *p* < 0.05 (Duncan’s multiple range test) (**B**) Transient expression of the *GUS* gene controlled by *pMuLRR-RLK* and *pMuLRR-RLK-AS* in *N. benthamiana* leaves, respectively. Tobacco leaves that were infiltrated were sampled at 6 h after SA treatments and at 36 h after *Pst*. DC3000 inoculation, respectively. Pst indicates *Pst*. DC3000.

To analyze the induced expression patterns of *MuLRR-RLK* and *MuLRR-RLK-AS*, the mulberry seedlings were challenged with *Pst.* DC3000 or SA, respectively. qRT-PCR analysis showed that the expression level of *MuLRR-RLK* was increased in the leaves challenged by *Pst.* DC3000 or SA. However, the expression level of *MuLRR-RLK-AS* was decreased in the leaves challenged (Figure 8A). Meanwhile, the putative promoters of the *MuLRR-RLK* and *MuLRR-RLK-AS* were cloned and submitted to the PlantCARE database to detect their cis-elements. The results showed that besides the common TATA-boxes and basic cis-acting elements, both *pMuLRR-RLK-AS* and *pMuLRR-RLK* contain various abiotic and biotic stress-responsive elements, including ABRE elements, MBS elements, TCA elements, G-boxes, ARE, Box4, P-boxes, TATC-boxes, etc. (Table 3 and Table 4). In addition, the *pMuLRR-RLK* or *pMuLRR-RLK-AS* was fused with *GUS* and transiently expressed in the tobacco leaves. Staining results showed that the *GUS* gene driven by *pMuLRR-RLK* was induced after inoculation of *Pst*. DC3000 or SA treatment (Figure 8B), while the *GUS* gene driven by *pMuLRR-RLK-AS* was repressed after inoculation of *Pst*. DC3000 or SA treatment. These data indicated that *MuLRR-RLK* and *MuLRR-RLK-AS* may be involved in mulberry resistance to pathogens, and SA may modulate their expression in mulberry.

### 3.6. MuLRR-RLK-AS Represses the Expression of MuLRR-RLK

**Figure 9:**
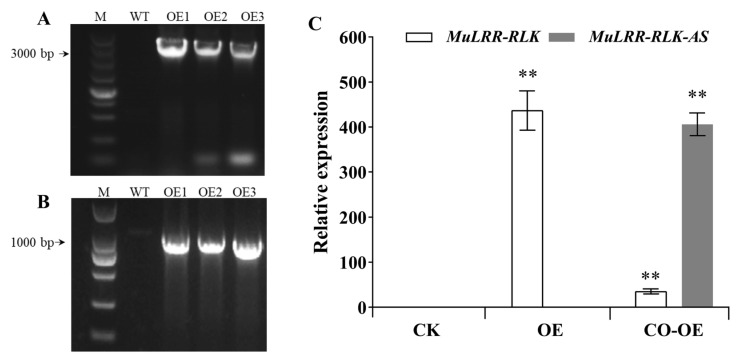
Expression levels of *MuLRR-RLK* in the transgenic Arabidopsis plants overexpressing *MuLRR-RLK* and co-expressing *MuLRR-RLK* and *MuLRR-RLK-AS*. (**A**,**B**) Genome PCR analysis showing that the *MuLRR-RLK* and *MuLRR-RLK-AS* genes were integrated into the genomes of transgenic Arabidopsis plants. (**C**) Expression levels of *MuLRR-RLK* in the transgenic Arabidopsis plants overexpressing *MuLRR-RLK* and co-expressing *MuLRR-RLK* and *MuLRR-RLK-AS*. The gene expression levels were evaluated via the ΔCt method with the *EF1-α* gene as a reference gene. Assays were performed three times with three replicates each time. Values are given as the mean ± SD of three experiments in each group. The double asterisks indicate significant differences at *p* < 0.05. CK indicates the transgenic empty vector plant. OE indicates the transgenic *MuLRR-RLK* plant. CO-OE indicates the co-expressing *MuLRR-RLK* and *MuLRR-RLK-AS* Arabidopsis plant.

Because an efficient regeneration system has not yet been established in mulberry, it is difficult to obtain transgenic mulberry trees. The nucleotide sequence identity of *MuLRR-RLK* and its homologue in Arabidopsis was low (74.6%). To verify the suppression of *MuLRR-RLK-AS* on the expression of *MuLRR-RLK,* the expression vector of *MuLRR-RLK* and the vector co-expressing *MuLRR-RLK* and *MuLRR-RLK-AS* were constructed, respectively, and transgenic Arabidopsis plants overexpressing *MuLRR-RLK* (OE) or co-expressing *MuLRR-RLK* and *MuLRR-RLK-AS* (CO-OE) were generated. The results of genome PCR analysis showed that both *MuLRR-RLK* and *MuLRR-RLK-AS* genes were integrated into the Arabidopsis genome (Figure 9A,B). Moreover, qRT−PCR analysis results indicated that the *MuLRR-RLK* gene was successfully expressed in the OE plants, and the expression levels of *MuLRR-RLK* were significantly higher in the OE Arabidopsis than those in the WT plants (Figure 9C). Although the expression levels of *MuLRR-RLK-AS* were significantly higher in the CO-OE plants than those in the WT plants, the expression level of *MuLRR-RLK* was significantly lower than that in the OE plants. This confirmed that the expression of *MuLRR-RLK-AS* effectively represses the expression of *MuLRR-RLK*.

### 3.7. MuLRR-RLK-AS Is a Negative Regulator of Plant Disease Resistance

**Figure 10:**
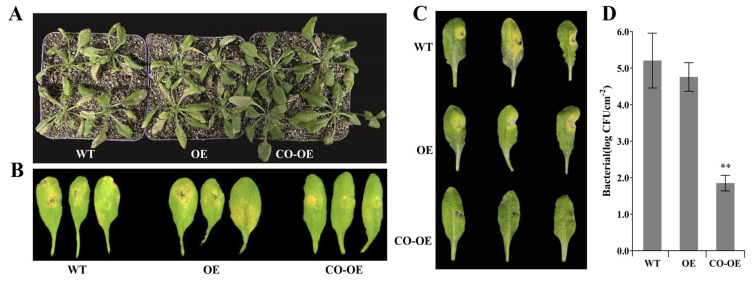
Negative regulation of *MuLRR−RLK−AS* on plant disease resistance to *B. cinerea* and *Pst*. DC 3000. (**A**) Symptoms on the leaves inoculated with *B. cinerea* observed 4 DAI. (**B**,**C**) Symptoms on the detached Arabidopsis leaves infiltrated with *Pst*. DC3000 observed 3 DAI. (**D**) Bacterial populations in the Arabidopsis leaves inoculated *Pst*. DC3000. Three independent experiments were analyzed 36 h after inoculation, with three replicates each time. Values in bar graphs are given as the mean ± SD of three experiments.The double asterisks indicate significant differences at *p* < 0.05. CK: Transgenic empty vector Arabidopsis seedlings. OE: Transgenic *MuLRR−RLK* Arabidopsis seedlings. CO−OE: Arabidopsis seedlings co−expressing *MuLRR−RLK* and *MuLRR−RLK−AS*.

Firstly, the CK, OE, and CO-OE Arabidopsis plants obtained above were challenged by *B. cinerea* to explore the possible role of *MuLRR-RLK-AS* in response to fungal pathogen infection. Four DAI, obviously expanding necrotic lesions were observed around the inoculated sites on the inoculated leaves of CK and CO-OE plants. In contrast, only mild disease symptoms could be observed on the surface of the inoculated leaves of OE plants (Figure 10A). In addition, the CK, OE, and CO-OE Arabidopsis plants were inoculated with *Pst.* DC3000 to explore the roles of *MuLRR-RLK-AS* in the response to pathogen infection. Three days after inoculation (DAI), the challenged leaves of CK and CO-OE plants showed obvious grayish brown lesion with chlorosis around the inoculation sites, especially the samples of CK showing severe disease symptoms. In contrast, no obvious disease symptoms were observed in the leaves of OE plants, although occasional mild chlorosis or necrosis was observed (Figure 10B,C). In addition, the bacterial populations of *Pst.* DC3000 strains in the inoculated leaves were determined, and the results showed that the strain number in the OE plants leaves was the lowest. Although the strain number in the CO-OE leaves was lower than that in the CK leaves, it was still significantly higher than those in the OE leaves (Figure 10D). This is consistent with the symptom phenotype of the leaves, indicating that overexpression of *MuLRR-RLK-AS* may inhibit the expression of *MuLRR-RLK*, weakening the resistance of the plant to *Pst.* DC3000. These results indicate that overexpression of *MuLRR-RLK* in Arabidopsis enhanced resistance to *Pst.* DC3000 and *B. cinerea*, while *MuLRR-RLK-AS* weakens plant disease resistance by repressing the expression of *MuLRR-RLK*.

### 3.8. Ectopic Expression of MuLRR-RLK Affects the Expression of Defense-Related Genes

**Figure 11:**
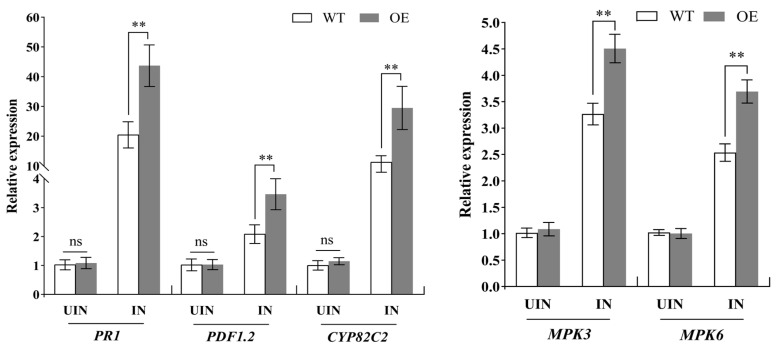
Regulation of *MuLRR-RLK* in the expression of defense-related genes, *MPK3*, and *MPK6*. With *EF1-α* as a reference gene, the gene expression levels were detected by qRT-qPCR using the comparative Ct method. The experiment was conducted three times, repeated three times each time, and the values in the bar graphs are the mean ± SD of three experiments. The double asterisk above the bar indicates significant difference at *p* < 0.05 according to Student’s *t*-test, and ns above the bar indicates no significant difference (*p* > 0.05). WT: Wild-ype Arabidopsis seedlings. OE: Transgenic *MuLRR-RLK* Arabidopsis seedlings. IN and UIN indicate the plants with or without pathogen inoculation, respectively.

According to the above results, *MuLRR-RLK-AS* weakens plant disease resistance by repressing the expression of *MuLRR-RLK.* To explore why the *MuLRR-RLK* gene was involved in plant disease resistance, the expression change in some defense-related genes in the transgenic *MuLRR-RLK* plants was evaluated. The results showed that there was no significant difference in the expression levels of the pathogenesis-related protein 1 gene (*PR-1*), plant defensin gene (*PDF1.2*), and cytochrome P450 protein gene (*CYP82C2*) between wild-type and transgenic *MuLRR-RLK* plants, and their expression levels were all very low. This indicates that overexpression of the *MuLRR-RLK* gene in Arabidopsis may not affect the basic expression levels of these defense-related genes without pathogen inoculation. However, it was found that 24 h post-inoculation with *Pst*. DC3000, the expression levels of *PR-1*, *PDF1.2*, and *CYP82C2* in the transgenic *MuLRR-RLK* Arabidopsis plants were higher than those in the wild-type plants (Figure 11). Since the *MPK3* and *MPK6* genes have been shown to have important roles in plant innate immune responses, to determine whether *MuLRR-RLK* affects the expression of the two genes, their expression levels in the *MuLRR-RLK*-overexpressing and wild-type Arabidopsis were measured. Similar to the expression patterns of the disease resistance-related genes mentioned above, there was no significant difference in the expression levels of *MPK3* and *MPK6* genes between the wild-type and transgenic *MuLRR-RLK* plants without pathogen inoculation. However, the expression levels of the two genes in transgenic plants were significantly higher than those in the wild-type plants when the plants were inoculated with *Pst.* DC3000 (Figure 11). These results indicate that when plants are infected with pathogens, *MuLRR-RLK* may play an important role in the disease resistance response by activating the MAPK cascade reaction, regulating the expression of defense genes.

## 4. Discussion

### 4.1. LncRNAs Are Involved in Regulating Gene Expression in Both Cis- and Trans-Manners in Response to Phytoplasma Infection

Recently, lncRNAs have been recognized as important regulators of plant responses to biotic and abiotic stresses [52]. In this study, an RNA-seq approach was used to investigate the transcriptomic changes in response to phytoplasma infection in mulberry, and 1794 novel lncRNAs were identified. To our knowledge, this is the first work to globally identify the lncRNAs responsive to phytoplasma infection in mulberry, and the results provided here can be useful for future research in this direction.

Previous studies have shown that lncRNAs, as cis- and trans-regulators, may regulate gene expression at both the transcriptional and posttranscriptional levels in various ways [53]. Cis-acting lncRNAs usually regulate the transcription of genes in close genomic proximity by recruiting or displacing transcription factors at the promoters of the neighboring genes [54]. To explore the potential roles of the cis-acting lncRNAs in response to phytoplasma infection in mulberry, an integrated DE lncRNA and coding gene analysis was performed to identify the cis-regulatory networks of lncRNAs, and 68 lncRNA-mRNA pairs were found. Furthermore, it was found that there were 37 lncRNAs with a positive correlation with their neighboring mRNAs, while only 31 neighboring lncRNA–mRNA pairs were found to be negatively correlated (Table 1). This suggests that the lncRNAs can cis-regulate the neighboring protein-coding genes in both positive and negative ways in response to phytoplasma infection in mulberry. However, the mechanism of the relationship between lncRNA–mRNA pairs needs to be further studied.

In addition to cis-regulatory roles, lncRNAs transcribed from an opposite strand of sense RNA in the same genomic regions (known as natural antisense transcript, lncNAT) may regulate the expression of sense RNA as trans-regulators by transcriptional or post-transcriptional mechanisms [13,55]. In this study, 44 DE mRNA–lncNAT pairs were identified (Table 2). Although the negative regulatory effects of lncNATs on the coding genes are more expectable, there were only nine DE lncNATs. Their target transcripts showed the opposite expression tendency, and the expression of most of DE lncNATs positively correlates with the expression of their targets (Table 2). It was suggested that some plant lncNATs can facilitate DNA methylation, histone modifications, chromatin conformation changes, and eventually up-regulate or down-regulate the transcription of their sense genes at the translational level. In addition, some plant lncNATs may affect mRNA decay by nucleases, mask miRNA binding sites, modulate protein translation, or produce endogenous siRNAs to execute RNA interference at the post-transcription level [22,56,57,58,59]. Therefore, further studies are needed to understand the action mechanism of these lncNATs.

Furthermore, GO enrichment analysis of the targeted genes of these DE cis- and trans-acting lncRNAs indicated that they were associated with various biological processes, such as the metabolic process, signal transduction, development, transcription regulatory, etc. (Supplementary Tables S3 and S4). Therefore, the DE lncRNAs may play important roles in diverse biologic processes, and their regulatory networks involved in the response to phytoplasma infection in mulberry are intricate. On one hand, the changes in these DE lncRNAs may disturb the expression of the genes involved in the normal growth and developmental and metabolic processes, resulting in the symptoms of shoot proliferation, witches’-broom, yellowing of the leaves, etc. On the other hand, the changes in DE lncRNAs may alter the expression of some resistance genes, thereby improving the resistance of mulberry to phytoplasma. Further experiments are required to uncover the action mechanism of these lncRNA–mRNA pairs that modulate the response of mulberry to phytoplasma.

### 4.2. MuLRR-RLK-AS Plays an Important Role in Regulating MuLRR-RLK Gene Expression in Response to Phytoplasma Infection

Leucine-rich repeat receptor-like protein kinases (LRR-RLKs) comprise one of the largest groups of receptor-like protein kinases (RLKs), which serve as receptors for signaling transduction pathways in plants [60,61]. It was suggested that LRR-RLKs are highly conserved and usually contain an extracellular, tandemly organized LRR domain, a single-pass transmembrane domain, and a functional protein kinase domain [62]. Our study showed that MuLRR-RLK protein structure has a high homology to other LRR-RLK proteins (Figure 5), indicating that it might have similar biological functions as other LRR-RLK proteins in the same subfamilies. It was reported that LRR-RLKs play vital roles in plant growth, development, and the responses to environmental stress, and some LRR-RLKs are associated with plant resistance to bacterial and fungal pathogen invasion [63,64,65]. However, to our knowledge, this is the first report that LRR-RLK genes are associated with the response to phytoplasma infection. In this study, *MuLRR-RLK* was identified as a phytoplasma-responsive gene. In addition, our data showed that although there was no significant difference in the expressions of PTI-related and *MAPK* genes between transgenic *MuLRR-RLK* and wild-type Arabidopsis plants, the expression levels of these genes in the transgenic plants were significantly higher than those in wild-type plants when inoculated with *Pst.* DC3000 (Figure 11). Meanwhile, our results showed that the MuLRR-RLK protein is localized on the plasma membrane (Figure 7B), and ectopic overexpression of the *MuLRR-RLK* gene confers the transgenic Arabidopsis plants with enhanced disease tolerance to *Pst.* DC3000 and *B. cinerea* (Figure 10). These results indicated that *MuLRR-RLK* might be involved in sensing signals arising from biotic stress and regulating defense responses, and the expression of *MuLRR-RLK* may be a strategy to overcome phytoplasma in diseased mulberry plants. Further research is needed to determine the molecular mechanism by which *MuLRR-RLK* senses the signals triggered by phytoplasma infection and mediates the resistance response to phytoplasma in mulberry.

Our results indicate that the expression of the *MuLRR-RLK* gene and the activity of its promoter are both induced by SA, while SA inhibits the expression of the *MuLRR-RLK-AS* gene and the activity of its promoter (Figure 8). It was reported that the levels of endogenous SA in the phytoplasma-infected plants are significantly increased [66]. Therefore, the increase in endogenous SA in the phytoplasma-infected leaves not only enhances the transcriptional activity of the promoter of *MuLRR-RLK* and increases the expression of *MuLRR-RLK*, but also reduces the post-transcriptional inhibitory effects of *MuLRR-RLK-AS* on *MuLRR-RLK* by inhibiting the expression of *MuLRR-RLK-AS*, ultimately leading to an increase in the expression of *MuLRR-RLK*. In the absence of pathogen infection, the SA level in plants is very low, and the expression level of *MuLRR-RLK-AS* may be higher, which inhibits the expression of *MuLRR-RLK* and thus maintains the expression of disease resistance-related genes at a low level. This may be beneficial for maintaining normal plant growth and development. Therefore, the SA level in plants may be an important factor in balancing the expression levels of *MuLRR-RLK-AS* and *MuLRR-RLK*, and the regulation of *MuLRR-RLK-AS* on the expression of *MuLRR-RLK* mediated by SA may be an important resistance response of mulberry to phytoplasma infection. Further research is needed to elucidate the exact mechanism behind this process.

## 5. Conclusions

In conclusion, the dynamic expression profiles of lncRNAs during the response to phytoplasma infection in mulberry were explored using high-throughput sequencing, and the DE lncRNAs and their target genes were identified. Moreover, the roles of the DE lncRNAs were discussed. Our results proved that *MuLRR-RLK-AS* acting as a trans-regulator can regulate the expression of *MuLRR-RLK*, which is a positive regulator of mulberry resistance. The information provided is particularly useful for understanding the functions and mechanisms of lncRNAs during the response to phytoplasma infection in mulberry.

## Figures and Tables

**Table 1:** Association analysis of the differentially expressed lncRNAs and their co-localization differentially expressed target genes.

LncRNAs				Up/Down Stream	Co-Localization Differentially Expressed Target Genes				
ID	log2FoldChange (IL/HL)	*p* Value	Up/Down		ID	log2FoldChange (IL/HL)	*p* Value	Up/Down	Description
XLOC_000009	−1.659904691	2.01 × 10^−5^	Down	Upstream_2k	XP_010086539.1	−6.285402219	9.71 × 10^−11^	Down	Uncharacterized protein LOC21384651
XLOC_003582	−1.357748564	4.19 × 10^−63^	Down	Downstream_2k	XP_010089960.1	1.622764533	2.69 × 10^−30^	Up	NADP-dependent glyceraldehyde-3-phosphate dehydrogenase
XLOC_004104	2.30176161	0	Up	Upstream_2k	XP_010090441.1	−1.498805857	8.26 × 10^−9^	Down	NAC domain containing protein 50 isoform X1
XLOC_004314	−11.62763875	4.50 × 10^−161^	Down	Downstream_2k	XP_010090692.1	5.044394119	5.83 × 10^−8^	Up	Uncharacterized protein
XLOC_005352	−1.359639818	7.41 × 10^−74^	Down	Downstream_2k	XP_010091710.1	−1.75161237	0.00017364	Down	Hypothetical protein
XLOC_005353	−2.142379817	1.19 × 10^−54^	Down	Downstream_2k	XP_010091710.1	−1.75161237	0.00017364	Down	Hypothetical protein
XLOC_006216	−8.087982542	0.000355214	Down	Downstream_2k	XP_010092498.1	11.06173466	0	Up	Hypothetical protein
XLOC_006461	−2.220725888	3.24 × 10^−11^	Down	Upstream_2k	XP_010092702.1	−2.115477217	8.81 × 10^−8^	Down	Pentatricopeptide repeat-containing protein
XLOC_006690	−1.496801279	2.07 × 10^−6^	Down	Downstream_2k	XP_010092905.1	1.325507985	2.24 × 10^−35^	Up	Common plant regulatory factor 1
XLOC_006961	−1.32577553	4.79 × 10^−5^	Down	Upstream_2k	XP_010093164.1	−2.191426071	1.03 × 10^−19^	Down	Hypothetical protein
XLOC_007096	−1.048521438	2.67 × 10^−8^	Down	Downstream_2k	XP_010093303.1	−1.415037499	1.18 × 10^−5^	Down	ATP-dependent zinc metalloprotease FtsH
XLOC_008373	2.902444746	5.16 × 10^−6^	Up	Upstream_2k	XP_010094597.1	1.378394431	4.77 × 10^−91^	Up	4-coumarate--CoA ligase-like 5
XLOC_008398	−2.45656083	0.474176	Down	Upstream_2k	XP_010094597.1	1.378394431	4.77 × 10^−91^	Up	4-coumarate--CoA ligase-like 5
XLOC_016866	−1.108741537	3.09 × 10^−6^	Down	Downstream_2k	XP_010094598.1	−1.708344916	7.10 × 10^−19^	Down	Protein ROOT PRIMORDIUM DEFECTIVE 1
XLOC_009663	−2.043818661	1.86 × 10^−23^	Down	Upstream_2k	XP_010095745.1	−2.172404286	9.14 × 10^−119^	Down	Pyruvate kinase isozyme A
XLOC_011123	−1.699511675	1.81 × 10^−14^	Down	Downstream_2k	XP_010097263.1	−1.570240995	7.69 × 10^−21^	Down	Hypothetical protein
XLOC_011929	1.009110152	2.46 × 10^−50^	Up	Downstream_2k	XP_010097937.1	−1.653442239	6.36 × 10^−61^	Down	Retrovirus-related Pol polyprotein from transposon TNT 1–94
XLOC_011968	−3.450165156	0.000183881	Down	Downstream_2k	XP_010097994.1	−6.087462841	0.00017296	Down	GDT1-like protein 2
XLOC_012270	−1.271836651	1.07 × 10^−23^	Down	Upstream_2k	XP_010098256.1	−1.115122616	1.46 × 10^−36^	Down	DEAD-box ATP-dependent RNA helicase ISE2
XLOC_012337	−2.438980184	0.474176	Down	Upstream_2k	XP_010098340.1	−2.850856561	0.00017039	Down	Zinc finger CCCH domain-containing protein 58
XLOC_012366	−2.450477186	0.0266538	Down	Upstream_2k	XP_010098340.1	−2.850856561	0.00017039	Down	Zinc finger CCCH domain-containing protein 58
XLOC_014320	−2.086989256	5.76 × 10^−12^	Down	Downstream_2k	XP_010100223.1	−1.047305715	1.35 × 10^−7^	Down	Ubiquitin carboxyl-terminal hydrolase 10
XLOC_014820	−1.871361676	6.68 × 10^−98^	Down	Downstream_2k	XP_010100733.1	−4.037089319	0.00018388	Down	Hypothetical protein
XLOC_015257	−2.853283109	0.474176	Down	Upstream_2k	XP_010101095.1	−1.461049897	2.36 × 10^−7^	Down	Histone-lysine N-methyltransferase
XLOC_015724	12.61184784	4.13 × 10^−69^	Up	Downstream_2k	XP_010101540.1	−2.722049907	0	Down	GDSL esterase/lipase
XLOC_017908	−1.9200952	1.66 × 10^−142^	Down	Upstream_2k	XP_010103622.1	−1.972293071	6.04 × 10^−21^	Down	Dihydroorotate dehydrogenase (quinone)
XLOC_018202	−1.743622263	5.76 × 10^−133^	Down	Upstream_2k	XP_010103913.1	−1.545434137	3.59 × 10^−9^	Down	Hypothetical protein
XLOC_018332	2.51571606	0.000105589	Up	Downstream_2k	XP_010104046.1	−1.906890596	9.50 × 10^−8^	Down	Hypothetical protein
XLOC_018333	1.508625478	2.35 × 10^−15^	Up	Downstream_2k	XP_010104046.1	−1.906890596	9.50 × 10^−8^	Down	Hypothetical protein
XLOC_018334	1.136596235	1.14 × 10^−12^	Up	Downstream_2k	XP_010104046.1	−1.906890596	9.50 × 10^−8^	Down	Hypothetical protein
XLOC_018352	1.274845329	8.73 × 10^−16^	Up	Upstream_2k	XP_010104047.1	−1.465663572	4.78 × 10^−11^	Down	Glucuronoxylan 4-O-methyltransferase 1
XLOC_018600	1.232238957	0.000128139	Up	Downstream_2k	XP_010104288.1	−1.080959321	2.26 × 10^−9^	Down	Uncharacterized protein LOC21388859
XLOC_018675	−1.476521201	4.78 × 10^−16^	Down	Upstream_2k	XP_010104373.1	−1.862496476	0.00022881	Down	Two-component response regulator-like protein
XLOC_018680	−2.179955771	2.14 × 10^−6^	Down	Upstream_2k	XP_010104373.1	−1.862496476	0.00022881	Down	Two-component response regulator-like protein
XLOC_018692	−2.139247136	2.33 × 10^−37^	Down	Upstream_2k	XP_010104373.1	−1.862496476	0.00022881	Down	Two-component response regulator-like protein
XLOC_018696	12.2276009	3.01 × 10^−28^	Up	Upstream_2k	XP_010104373.1	−1.862496476	0.00022881	Down	Two-component response regulator-like protein
XLOC_019166	−1.120989021	2.10 × 10^−94^	Down	Downstream_2k	XP_010104915.1	−1.100273908	1.37 × 10^−28^	Down	Uncharacterized protein LOC21406865
XLOC_019555	−2.58203706	0.0595904	Down	Downstream_2k	XP_010105201.1	−5.087462841	2.00 × 10^−5^	Down	Sn1-specific diacylglycerol lipase beta
XLOC_019810	14.10733152	1.15 × 10^−47^	Up	Downstream_2k	XP_010105413.1	−1.70571466	2.40 × 10^−31^	Down	Putative GDP-L-fucose synthase 2
XLOC_020110	−1.030050297	2.09 × 10^−24^	Down	Upstream_2k	XP_010105705.1	−1.650322233	8.17 × 10^−45^	Down	Guanylate kinase 2
XLOC_020112	1.243959167	7.39 × 10^−25^	Up	Upstream_2k	XP_010105705.1	−1.650322233	8.17 × 10^−45^	Down	Guanylate kinase 2
XLOC_020267	−1.009344657	8.55 × 10^−6^	Down	Downstream_2k	XP_010105833.1	−2.321928095	1.57 × 10^−10^	Down	Pre-mRNA-processing protein 40A isoform X2
XLOC_020549	−3.468308658	2.93 × 10^−10^	Down	Upstream_2k	XP_010106108.1	−7.6794801	8.38 × 10^−20^	Down	Hypothetical protein
XLOC_020799	−3.174801049	8.27 × 10^−9^	Down	Upstream_2k	XP_010106332.1	4.558967292	1.88 × 10^−156^	Up	Hypothetical protein
XLOC_020823	−1.290572462	9.68 × 10^−10^	Down	Upstream_2k	XP_010106349.1	−3.019590728	1.52 × 10^−41^	Down	Hypothetical protein
XLOC_020815	−1.550204751	4.40 × 10^−5^	Down	Downstream_2k	XP_010106354.1	2.674712213	1.36 × 10^−243^	Up	G-type lectin S-receptor-like serine/threonine-protein kinase
XLOC_021165	−1.220913717	3.65 × 10^−8^	Down	Upstream_2k	XP_010106679.1	1.85708704	4.53 × 10^−17^	Up	Inorganic pyrophosphatase 1
XLOC_021166	−2.999715655	2.63 × 10^−30^	Down	Upstream_2k	XP_010106679.1	1.85708704	4.53 × 10^−17^	Up	Inorganic pyrophosphatase 1
XLOC_021967	−1.459141927	3.89 × 10^−9^	Down	Downstream_2k	XP_010107456.1	−3.378511623	2.13 × 10^−13^	Down	uncharacterized protein LOC21385704 isoform X1
XLOC_022178	−1.074733339	1.69 × 10^−54^	Down	Downstream_2k	XP_010107670.1	1.48112669	2.62 × 10^−11^	Up	probable indole-3-acetic acid-amido synthetase GH3.6
XLOC_023091	2.645694109	1.02 × 10^−6^	Up	Downstream_2k	XP_010108540.1	−2.264499815	3.96 × 10^−15^	Down	hypothetical protein
XLOC_024046	−2.458590043	0.230884	Down	Downstream_2k	XP_010109450.1	−1.638260727	1.48 × 10^−60^	Down	mitochondrial carrier protein MTM1 isoform X1
XLOC_024072	−1.363720387	1.03 × 10^−15^	Down	Downstream_2k	XP_010109466.1	−1.469695084	8.90 × 10^−40^	Down	hypothetical protein
XLOC_024250	1.511943985	4.91 × 10^−15^	Up	Downstream_2k	XP_010109645.1	1.030214613	9.23 × 10^−51^	Up	transcription factor DIVARICATA
XLOC_024279	−2.968724068	0.474176	Down	Upstream_2k	XP_010109661.1	−2.164282855	1.62 × 10^−187^	Down	pentatricopeptide repeat-containing protein
XLOC_024407	−1.218449856	1.04 × 10^−22^	Down	Upstream_2k	XP_010109786.1	−1.513069582	3.18 × 10^−11^	Down	uncharacterized protein At4g10930
XLOC_025437	−2.561579471	0.474176	Down	Downstream_2k	XP_010110803.1	1.12963528	2.27 × 10^−5^	Up	RING finger protein B
XLOC_025452	−2.474769468	0.474176	Down	Upstream_2k	XP_010110804.1	5.720122084	8.40 × 10^−129^	Up	leucoanthocyanidin reductase
XLOC_025453	3.77129058	6.73 × 10^−9^	Up	Upstream_2k	XP_010110804.1	5.720122084	8.40 × 10^−129^	Up	leucoanthocyanidin reductase
XLOC_025463	1.448311774	5.88 × 10^−9^	Up	Upstream_2k	XP_010110804.1	5.720122084	8.40 × 10^−129^	Up	leucoanthocyanidin reductase
XLOC_025605	1.268119766	1.27 × 10^−140^	Up	Downstream_2k	XP_010110915.1	−1.776621795	1.26 × 10^−42^	Down	hypothetical protein
XLOC_025845	1.086054103	0.000228706	Up	Upstream_2k	XP_010111144.1	−5.584962501	2.31 × 10^−6^	Down	hypothetical protein
XLOC_025906	1.313631149	7.94 × 10^−25^	Up	Upstream_2k	XP_010111198.1	−1.252187024	3.51 × 10^−8^	Down	putative E3 ubiquitin-protein ligase RING1a isoform X2
XLOC_025907	2.290116081	5.36 × 10^−23^	Up	Upstream_2k	XP_010111198.1	−1.252187024	3.51 × 10^−8^	Down	putative E3 ubiquitin-protein ligase RING1a isoform X2
XLOC_025909	−1.440184589	9.82 × 10^−6^	Up	Upstream_2k	XP_010111198.1	−1.252187024	3.51 × 10^−8^	Down	putative E3 ubiquitin-protein ligase RING1a isoform X2
XLOC_026031	−1.440184589	9.82 × 10^−6^	Down	Downstream_2k	XP_010111442.1	1.015266757	0.0001083	Up	hypothetical protein
XLOC_026652	−2.355017768	0.474176	Down	Upstream_2k	XP_010111922.1	−2.30256277	1.38 × 10^−15^	Down	uncharacterized protein LOC21393124
XLOC_026790	−1.123960181	9.54 × 10^−5^	Down	Upstream_2k	XP_010112073.1	−2.692490965	1.52 × 10^−20^	Down	ATP-dependent Clp protease ATP-binding subunit ClpX

**Table 2:** Association analysis of the differentially expressed lncRNAs and their differentially expressed target genes.

LncRNAs				Target Genes				
ID	log2FoldChange (B/J)	*p* Value	Up/Down	ID	log2FoldChange (BB/JJ)	*p* Value	Up/Down	Descrition
XLOC_001590	3.837441902	0.000471504	Up	XP_010088049.1	2.605901838	6.15 × 10^−43^	Up	Chaperone protein DnaJ
XLOC_003017	4.009664269	0.012449373	Up	XP_010089382.1	−1.15417093	2.37 × 10^−8^	Down	PAP-specific phosphatase HAL2-like protein
XLOC_003046	−2.433070931	0.014546012	Down	XP_010089404.1	−2.06667104	3.31 × 10^−9^	Down	Hypothetical protein L484_013795
XLOC_003503	−1.599653771	1.22743 × 10^−11^	Down	XP_010089852.1	−7.033423002	2.00 × 10^−5^	Down	Subtilisin-like protease
XLOC_004551	−1.779844507	0.034037713	Down	XP_010090841.1	−1.385653692	4.59 × 10^−6^	Down	Putative polygalacturonase
XLOC_004954	2.379663284	7.51328 × 10-^−5^	Up	XP_010091231.1	2.011383996	0	Up	Hypothetical protein L484_005255 ]
XLOC_005131	−2.31198078	0.003175704	Down	XP_010091411.1	−2.021479727	7.08 × 10^−11^	Down	Chlorophyll a-b binding protein 16
XLOC_005342	−8.732455766	2.79104 × 10^−56^	Down	XP_010091645.1	2.479780264	5.94 × 10^−26^	Up	UDP-glycosyltransferase 89A2
XLOC_006046	−1.595761698	2.7063 × 10^−13^	Down	XP_010092313.1	−2.662965013	1.18 × 10^−40^	Down	Putative galacturonosyltransferase 12
XLOC_006462	−2.022357412	4.62933 × 10^−36^	Down	XP_010092676.1	−1.69538649	2.14 × 10^−20^	Down	Hypothetical protein L484_019750
XLOC_009619	−1.020506348	6.48115 × 10^−7^	Down	XP_010095699.1	−1.156119202	2.20 × 10^−7^	Down	Putative endo-1,4-beta-xylanase C
XLOC_010285	−1.479609008	0.00017518	Down	XP_010096371.1	5.169925001	0.00017525	Up	Myb-related protein B
XLOC_011021	−2.356597415	1.18979 × 10^−10^	Down	XP_010097040.1	−2.938599455	1.17 × 10^−8^	Down	Hypothetical protein L484_003871
XLOC_011087	−1.189537055	1.92884 × 10^−8^	Down	XP_010097097.1	−1.310381065	0	Down	Hypothetical protein L484_019536
XLOC_011132	−1.325768866	3.97956 × 10^−11^	Down	XP_010097155.1	−1.830074999	7.78 × 10^−6^	Down	ATPase 9, plasma membrane-type
XLOC_011724	−1.892272481	7.40323 × 10^−13^	Down	XP_010097661.1	−1.523561956	4.05 × 10^−14^	Down	Phytosulfokine receptor 2
XLOC_012537	−1.437284073	0.002462778	Down	XP_010098515.1	−1.659924558	4.93 × 10^−27^	Down	Hypothetical protein L484_025954
XLOC_012821	−1.545482034	1.99649 × 10^−14^	Down	XP_010098738.1	−1.74723393	2.02 × 10^−8^	Down	Pectinesterase 3
XLOC_012923	1.151448857	5.66295 × 10^−6^	Up	XP_010098833.1	−1.27364808	3.01 × 10^−6^	Down	UDP-glycosyltransferase 73C3
XLOC_013301	−1.135788866	0.000554496	Down	XP_010099215.1	−1.963474124	3.86 × 10^−34^	Down	Hypothetical protein L484_010155
XLOC_013697	−2.587472054	9.67889 × 10^−17^	Down	XP_010099597.1	−1.17290886	5.38 × 10^−6^	Down	Histone H3-like centromeric protein
XLOC_015378	−1.534366505	0.000274527	Down	XP_010101197.1	−1.021061616	0.000108539	Down	Hypothetical protein L484_015001
XLOC_016174	−1.659968292	5.73067 × 10^−20^	Down	XP_010101961.1	−2.408084739	2.01 × 10^−5^	Down	Hypothetical protein L484_011978
XLOC_016448	9.017824501	0.008623184	Up	XP_010102287.1	5.984893108	1.89 × 10^−19^	Up	Hypothetical protein L484_024569
XLOC_017317	−2.24788776	1.18979 × 10^−10^	Down	XP_010103054.1	−2.815174427	7.64 × 10^−92^	Down	Hypothetical protein L484_001885
XLOC_017564	−1.780652222	5.73067 × 10^−20^	Down	XP_010103289.1	4.930737338	3.35 × 10^−92^	Up	3’-hydroxy-N-methyl-(S)-coclaurine 4’-O-methyltransferase
XLOC_018767	−2.14733932	8.58013 × 10^−35^	Down	XP_010104430.1	−4.087462841	0.000183881	Down	Hypothetical protein L484_016029
XLOC_019411	−1.597933878	0.006303773	Down	XP_010105051.1	−2.297266041	3.97 × 10^−15^	Down	Hypothetical protein L484_001492
XLOC_020739	−2.772398916	2.36945 × 10^−8^	Down	XP_010106301.1	−3.795859283	3.06 × 10^−10^	Down	Magnesium-transporting ATPase, P-type 1
XLOC_020789	1.043080361	4.3553 × 10^−7^	Up	XP_010106332.1	4.558967292	1.88 × 10^−156^	Up	Hypothetical protein L484_004731
XLOC_021217	−1.307207494	8.68908 × 10^−7^	Down	XP_010106725.1	−1.634715536	5.88 × 10^−5^	Down	Myb-related protein Myb4
XLOC_021596	−2.443238328	0.003175704	Down	XP_010107118.1	−1.093574115	9.06 × 10^−5^	Down	NAC domain-containing protein 1
XLOC_022461	−1.911411449	0.031545424	Down	XP_010107850.1	−2.275735285	7.51 × 10^−53^	Down	Hypothetical protein L484_027437
XLOC_022591	2.284500804	8.77802 × 10^−9^	Up	XP_010108041.1	1.83518913	1.25 × 10^−6^	Up	Exocyst complex component 7
XLOC_022840	1.471119577	0.008783891	Up	XP_010108284.1	−6.906890596	3.53 × 10^−19^	Down	Hypothetical protein L484_007137
XLOC_022990	−9.988102379	7.94406 × 10^−5^	Down	XP_010108424.1	2.03562391	8.43 × 10^−11^	Up	Nudix hydrolase 15
XLOC_025085	9.747083561	6.51127 × 10^−23^	Up	XP_010110394.1	1.094718657	0.000123589	Up	Hypothetical protein L484_022797
XLOC_025632	−2.675688053	7.45831 × 10^−12^	Down	XP_010110923.1	−1.80937409	2.02 × 10^−73^	Down	Omega-hydroxypalmitate O-feruloyl transferase
XLOC_026011	−2.847603572	7.57567 × 10^−8^	Down	XP_010111400.1	1.961110987	1.28 × 10^−25^	Up	Multiple C2 and transmembrane domain-containing protein 2
XLOC_026715	−2.99257368	2.63073 × 10^−53^	Down	XP_010111977.1	−3.89077093	2.45 × 10^−17^	Down	Putative peptide/nitrate transporter
XLOC_026802	−2.726806211	4.61238 × 10^−40^	Down	XP_010112045.1	−1.807354922	1.62 × 10^−8^	Down	Endoglucanase 9
XLOC_027017	−1.271269054	5.03723 × 10^−10^	Down	XP_010112258.1	−1.332575339	0.000230944	Down	MADS-box transcription factor 27
XLOC_027018	−2.185948672	1.18979 × 10^−10^	Down	XP_010112258.1	−1.332575339	0.000230944	Down	MADS-box transcription factor 27
XLOC_027445	−1.081848858	0.033465549	Down	XP_010112683.1	4.024384159	5.29 × 10^−30^	Up	Putative LRR receptor-like serine/threonine-protein kinase

**Table 3:** Cis-acting elements of the *pMuLRR-RLK-AS*.

Site Name	Number	Sequence	Function
ABRE	2	ACGTG	Cis-acting element involved in abscisic acid responsiveness
ACE	1	GACACGTATG	Cis-acting element involved in light responsiveness
ARE	1	AAACCA	Cis-acting regulatory element essential for anaerobic induction
ATCT-motif	1	AATCTAATCC	Part of a conserved DNA module involved in light responsiveness
AuxRR-core	1	GGTCCAT	Cis-acting regulatory element involved in auxin responsiveness
Box 4	1	ATTAAT	Part of a conserved DNA module involved in light responsiveness
CAAT-box	31	CCAAT	Common cis-acting element in promoter and enhancer regions
CAT-box	1	GCCACT	Cis-acting regulatory element related to meristem expression
GARE-motif	1	TCTGTTG	Gibberellin-responsive element
GATA-motif	1	GATAGGA	Part of a light responsive element
G-Box	3	TACGTG	Cis-acting regulatory element involved in light responsiveness
MBS	1	CGGTCA	MYB binding site involved in drought-inducibility
P-box	1	CCTTTTG	Gibberellin-responsive element
TATA-box	13	TATA	Core promoter element of approximately −30 transcription starts
TATC-box	1	TATCCCA	Cis-acting element involved in gibberellin-responsiveness
TCA-element	1	CCATCTTTTT	Cis-acting element involved in salicylic acid responsiveness
TCT-motif	1	TCTTAC	Part of a light responsive element
TGA-element	1	AACGAC	Auxin-responsive element

**Table 4:** Cis-acting elements of the *pMuLRR-RLK*.

Site Name	Number	Sequence	Function
ABRE	2	ACGTG	Cis-acting element involved in abscisic acid responsiveness
ARE	2	AAACCA	Cis-acting regulatory element essential for anaerobic induction
Box 4	1	ATTAAT	Part of a conserved DNA module involved in light responsiveness
CAAT-box	46	CCAAT	Common cis-acting element in promoter and enhancer regions
CAT-box	1	GCCACT	Cis-acting regulatory element related to meristem expression
CGTCA-motif	2	CGTCA	Cis-acting regulatory element involved in meja-responsiveness
G-Box	3	TACGTG	Cis-acting regulatory element involved in light responsiveness
GCN4_motif	1	TGAGTCA	Cis-regulatory element involved in endosperm expression
GT1-motif	1	GGTTAA	Light responsive element
LTR	1	CCGAAA	Cis-acting element involved in low-temperature responsiveness
MBS	1	CGGTCA	MYB binding site involved in drought-inducibility
P-box	1	CCTTTTG	Gibberellin-responsive element
O2-site	2	GATGA(C/T)(A/G)TG(A/G)	Cis-acting regulatory element involved in zein metabolism regulation
TATA-box	46	TATA	Core promoter element of approximately −30 transcription starts
TC-rich repeats	1	GTTTTCTTAC	Cis-acting element involved in defense and stress responsiveness
TCA-element	1	CCATCTTTTT	Cis-acting element involved in salicylic acid responsiveness
TCT-motif	1	TCTTAC	Part of a light responsive element

## Data Availability

The transcriptome data have been deposited to GenBank with the dataset code SRR28089553 and SRR28089552.

## Supplementary Materials

The following supporting information can be downloaded at: https://www.mdpi.com/article/10.3390/biom14030308/s1, Table S1. Differentially expressed genes between healthy and infected mulberry leaves. Table S2. Differentially expressed lnRNAs between healthy and infected mulberry leaves. Table S3. Association analysis of differentially expressed lncRNAs and their co-localization differentially expressed target genes. Table S4. Association analysis of the differentially expressed lncRNAs and their differentially expressed target genes.
